# Multidimensional intratumour heterogeneity in neuroblastoma

**DOI:** 10.18632/oncotarget.26524

**Published:** 2019-01-01

**Authors:** Kristoffer von Stedingk, David Gisselsson, Daniel Bexell

**Affiliations:** Daniel Bexell: Department of Laboratory Medicine, Division of Translational Cancer Research, Lund University, Lund, Sweden; Kristoffer von Stedingk: Division of Pediatric Oncology, Department of Clinical Sciences, Lund University, University Hospital, Lund, Sweden; Department of Oncogenomics, Amsterdam UMC, location AMC, University of Amsterdam, The Netherlands

**Keywords:** pediatric cancer, neuroblastoma, intratumour heterogeneity, clonal evolution, patient-derived xenograft (PDX)

Neuroblastoma (NB) is a solid childhood cancer originating from the sympathetic nervous system. Most NBs arise from the adrenal gland but metastases to distant sites such as bone marrow, liver and lungs are not uncommon. Despite intense treatment, including high-dose chemotherapy, many high-risk NBs relapse and treatment resistance is a significant clinical problem.

NB is a heterogeneous disease, i.e., tumours display high inter-tumour heterogeneity. Recent findings also point to genetic intratumour heterogeneity (ITH) in NB, albeit lower than has been observed in other adult tumours. This can be a result of clonal evolution due to Darwinian selection among tumour cells [1]. It can also emerge in the absence of strong selection through the mechanism of genetic drift [2]. ITH is associated to progressive disease and treatment resistance, likely because of selection of treatment resistant clones [3].

There are different dimensions of genetic ITH in NB. Temporal genetic ITH has been observed when comparing treatment naïve and matched relapsed NBs. Relapsed NBs are enriched in mutations (e.g., in *ALK*, *NRAS* genes) predicted to activate the RAS-MAPK pathway, Hippo-YAP pathway and epithelial-mesenchymal processes [4, 5]. These findings open up opportunities for therapeutic targeting of relapsed NB. However, a druggable mutation detected only in a relapse, not in the corresponding primary tumour, is unlikely to be present in all neoplastic cells in the patient’s body; it will not be part of the “trunk” of the tumour’s phylogenetic tree. Hence targeting relapse-specific mutations may only provide temporary remission. Recent reports have also shown that a high proportion (> 90%) of primary NBs exhibit spatial genetic ITH, i.e. distinct tumour cell genotypes in different regions of the same tumour [6]. The fact that mutations present in the major clone at relapse have their origin in subclones of the primary tumour demonstrates that spatial and temporal ITH are intimately connected. This is also highlighted by the fact that relapse tumours *per se* frequently exhibit spatial genetic variation [6].

The fluctuating presence of clones over space and time in NB is complex, but may nevertheless be a result of a limited number of underlying principles. In fact, spatiotemporal clonal evolution in paediatric tumours appears to be accounted for by four distinct evolutionary trajectories, including (1) regional fluctuations of subclones, (2) subclones that coexist over vast anatomic space, (3) clonal sweeps, where one clone expands to outgrow all other clones in a region, and (4) so-called evolutionary explosions, a type of massive regional chromosomal instability triggered by inactivation of the p53 pathway. Of these, the presence in the primary tumour of the two latter trajectories are strong predictors of high-risk disease and inferior outcome in NB as well as other paediatric cancers [6]. This indicates that evolvability is a core component in the resilience of NB cells against oncological therapy.

ITH is often described as genetic and functional differences across the neoplastic cells themselves, however heterogeneity of the tumour microenvironment is overlooked. Xenograft experiments using the same cell populations implanted in different environments display highly varying growth patterns. The same cells injected subcutaneously *versus* orthotopically display non-invasive localized tumour expansion *versus* metastatic spreading to distant organs, respectively [7]. This suggests that stromal composition plays an important role in regulating tumour cell behaviour. These observations are often conducted on a whole tumour-basis. Investigation into locoregional stromal ITH within a given tumour is also highly relevant and may play as important a role in tumour cell behaviour as the ITH of the tumour cells themselves. Indeed, it has been described that “undifferentiated” NB cells appear to locate themselves along vascularized niches within the tumours [8]. Delivery of oxygen to the tumour environment, a process largely controlled by the tumour stroma and vasculature, can lead to a hypoxic (shortage of oxygen) environment. This has been shown to dedifferentiate NB cells into a more immature stem cell-like state with increased aggressive characteristics [9].

In line with this, recent studies have also described multiple cell-type states of NB cells, termed *mesenchymal* (“*undifferentiated*”) and *adrenergic*, that exist within the same tumour. These cells are genetically identical however differ in their transcriptional and epigenetic landscape [10]. In a similar study conducted by Boeva et al. they again could identify subpopulations of tumour cells based on transcriptional and epigenetic diversity including sympathetic noradrenergic, neural-crest cell -like identity, and mixed-type [11]. Similar genetically identical, multi-differentiation state observations have also been made in melanoma, glioblastoma, lung cancer and breast cancer, amongst others, indicating the broad relevance of this phenomenon beyond just NB. Tumour cells in a mesenchymal state have been shown to be more chemo-resistant and are suggested as responsible for post-therapy relapse, pointing to clinically significant functional ITH in NB [10]. Mechanisms behind how these subtypes are selected for, however, remain elusive. It is likely that intratumour locoregional heterogeneity of the stroma may play a vital role in the regulation of the phenotype the tumour cells including their metastatic behaviour and response to therapy.

Using patient-derived orthotopic xenograft (PDOX) models of NB, we recently described additional evidence of functional ITH in NB. By implanting multiple NB samples derived from a single tumour into ten mice, we uncovered clear differences in transcriptional profiles and divergent tumour growth rates *in vivo*. This is despite the tumours being genetically identical. Furthermore, ontology analysis revealed differences in genes regulating neuronal/mesenchymal phenotypes as well as stromal and angiogenic processes, again indicating the involvement of tumour-stromal interactions in the ITH observed in NB [12].

**Figure 1:**
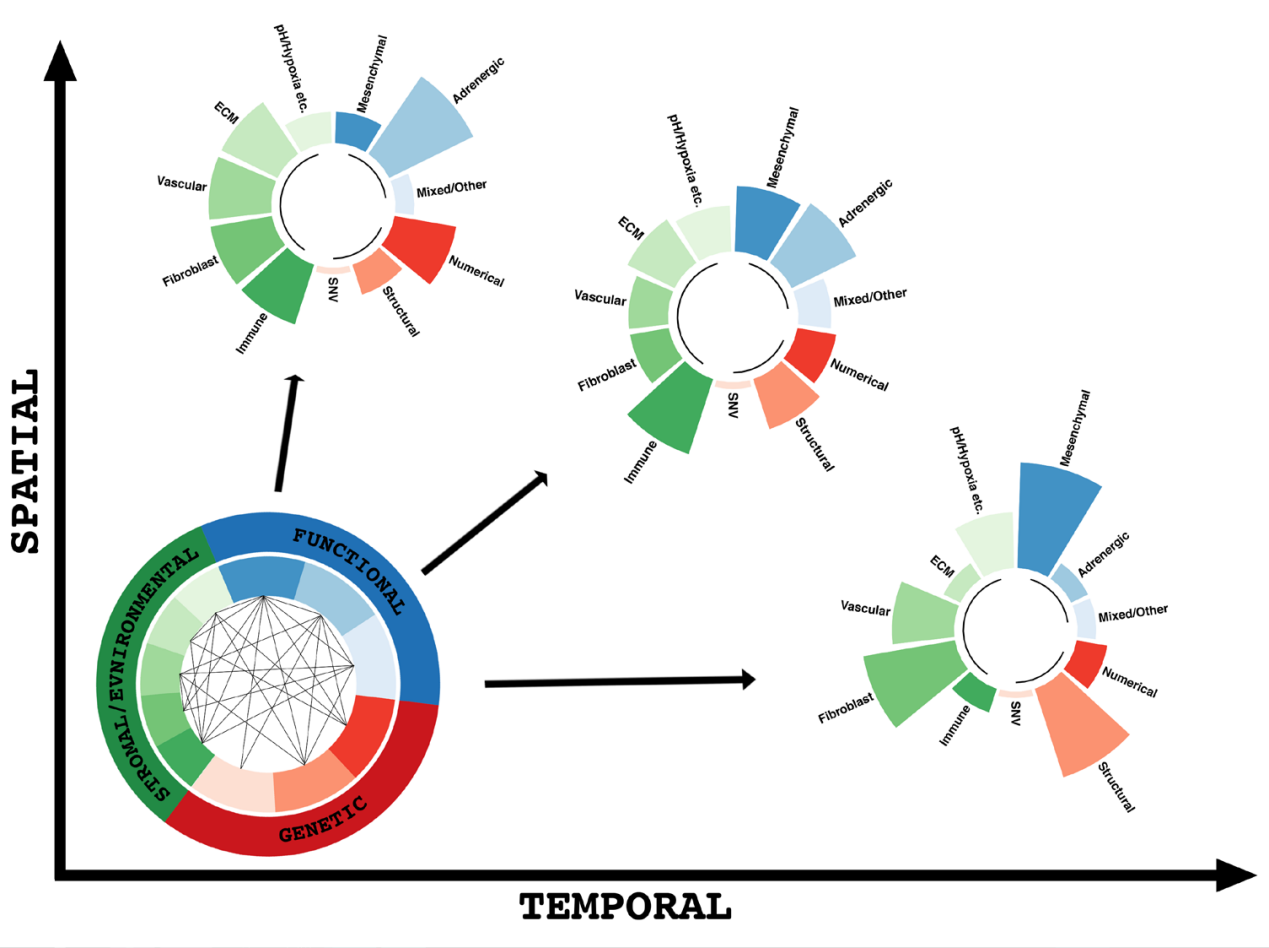
Multidimensional intratumour heterogeneity in neuroblastoma Within all solid tumours, including neuroblastoma, exists a complex composition of multiple cell types, which can differ genetically, functionally or environmentally. Multiple interactions between these cell types may influence their committed and/or plastic trajectories resulting in different cellular composition depending on where (spatially) or when (temporally) you examine the tumour. For example, one biopsy may show a “less aggressive” composition with mainly adrenergic tumour cells displaying numerical changes and a balanced stromal environment with a healthy level of immune infiltrate (top left). A different biopsy from the same patients from either a different region of the tumour (spatial variation), or a biopsy obtained at a later time point, for example after therapy, may display a “more aggressive” composition, with tumour cells that are phenotypically mesenchymal, harbouring structural genetic aberrations and an inhibited immune infiltration (bottom right). Along the axes of time and space, the heterogeneity and interplay between these cellular subtypes can vary, stressing the importance of considering multiple biopsies from the same patient as well as making sure that examined biopsies are temporally relevant for the patient in question. While spatial and temporal heterogeneity are the main axes on which all other heterogeneity changes and evolves, it should be noted that a temporal heterogeneity must stem from underlying spatial heterogeneity, while spatial heterogeneity require time to form. Investigating and understanding the complex interactions at play behind tumour heterogeneity will be essential for future pre-clinical modelling and therapy development. Single nucleotide variants (SNVs), although reported in neuroblastoma cases such as *ALK*-mutations, are relatively rare when compared to other cancers, and therefore only represent a small subset of the genetic heterogeneity within this schematic.

In summary, there are several dimensions of ITH in NB, including genetic and functional diversity of NB cells, as well as microenvironmental tumour heterogeneity, all of which can relate to progression and treatment response (Figure 1). Disentangling the interplay between these different ITH dimensions will be of utmost importance for improved preclinical modelling. ITH must also be considered during the design of novel therapeutic strategies for NB. For example, the finding of ITH for a number of clinical treatment biomarkers highlights the need to always base targeted treatment decisions on information from the latest available tumour biopsy from each patient.
